# Driver behaviour and traffic accident involvement among professional heavy semi-trailer truck drivers in China

**DOI:** 10.1371/journal.pone.0260217

**Published:** 2021-12-02

**Authors:** Wanli Han, Jianyou Zhao, Ying Chang

**Affiliations:** 1 Shanghai Urban Operation (Group) Co., Ltd, Shanghai, China; 2 College of Transportation Engineering, Chang’an University, Xi’an, Shaanxi, China; 3 School of Automobile, Chang’an University, Xi’an, Shaanxi, China; Universitat de Valencia, SPAIN

## Abstract

The purpose of this study was to develop a driving behavior scale for professional drivers of heavy semi-trailer trucks in China, and study the causes of such driving behavior and its impact on traffic safety operation. Data was processed by IBM SPSS 25. In addition to principal component analysis, Promax rotation, Bartlett’s test, Cronbach’s alpha, correlation analysis and binary logistic regression were examined. A DBQ with 4 dimensions and 20 items, and a PDBQ with 1 dimension and 6 items were developed for professional drivers of heavy semi-trailer trucks in China. The KMO coefficients of PDBQ and DBQ were 0.822 and 0.852, respectively, and the significant level of Bartlett’s popularity test was p < 0.0001. The accident prediction model showed that the variables related to traffic accidents were negligence/lapses and driving time of heavy semi-trailer truck drivers. 1–5 a.m. was found to be the most dangerous period for drivers of medium and heavy semi-trailer trucks, during which accidents were most likely to happen. As negligence/lapses increased by one unit, the probability of traffic accidents increased by 2.293 times.

## Introduction

With the advantages of large volume and flexible transportation, heavy semi-trailer truck played an important role in highway transportation, and was integral to highway freight industry. At present, the highway freight market was developing by virtue of heavy loading vehicles. However, the proportion of the death toll caused by heavy truck accidents to that caused by road traffic accidents in total was also on the rise. Compared with small vehicles, heavy trucks were large in model structure and poor in dynamic performance due to their heavy loading and inflexibility, thus consuming more road space-time resources in driving. This made them easy to cause major and serious traffic accidents. Traffic accidents involving heavy semi-trailer trucks had brought huge losses to public safety and social economy. As the main participant in freight industry, professional drivers of heavy semi-trailer trucks were one of the key factors of traffic safety. In the investigation of traffic accidents involving long-distance trucks, it was found that 90% of the accidents were caused by drivers [1]. Professional drivers of long-distance trucks experienced the monotony of long-distance driving and irregular routine. Compared with ordinary drivers, truck drivers assumed higher risks of traffic accidents. According to Lynn and Lockwood [2], it was found that company car drivers caused about 50% more accidents than ordinary drivers in terms of their higher mileages. Dimmer and Parker [3] also found that 27% of professional drivers reported involvement in traffic accidents over the last three years, compared to a level of 18% from the wider driving population. Therefore, it was of great significance to study the professional drivers of heavy trucks, especially the driving behavior and traffic accident prediction in respect of professional drivers of heavy semi-trailer trucks.

### Research status of driving behavior and its influencing factors

At present, there were lots of researches on driving behavior of professional and non-professional drivers, among which the application of the Driver Behavior Questionnaire (DBQ) to collect information about driving behavior of drivers in different types and the method of predicting drivers’ involvement in traffic accidents were proved effective [4–7]. More than 200 DBQ-based papers had been published since the DBQ with 3 factors (violations, dangerous errors, and harmless lapses) and 50 items was employed by Reason and Manstead [8]. Presently, most of the studies applied a DBQ structured with 4 factors and 27 or 28 items, which generally included ordinary violations, aggressive violations, errors and lapses [6]. However, the DBQ appeared to be primarily concerned with aberrant driving practices related to traffic safety. In recent years, some researchers added positive driving behavior factors into the DBQ, and found that the positive driving behaviors of drivers affected their aberrant driving behaviors and had an impact on traffic accidents [6,9–11].

Factor structures varied in different countries and depend on types of vehicles and drivers. Later studies showed that the main distinction between errors and violations seemed to occur in different populations in many countries [7,8,12–18]. Mallia and lazuras [19] used a 3-factor DBQ to study Italian bus drivers, and found that only violations were related to the accident risk of bus drivers. Marko et al. [10] researched on drivers who transported dangerous goods in Serbia. Factor analysis showed that the 5-factor structured DBQ was more suitable for the professional drivers who transported dangerous goods in Serbia. It was also found that the daily driving time (exposure) of the professional drivers transporting dangerous goods was positively related to inattention and violations, negatively related to positives, and directly related to the driver’s fatigue and error frequency. Milad et al. [18] used a 4-factor structured DBQ to study the direct and indirect effects of background variables and aberrant driving behavior of Irish drivers. It was found that violations, aggressive violations and errors of drivers were related to traffic accidents, while lapses were not related to traffic accidents. It was also found that age, driving age, education background, driving mileage, rest status, income and vehicle ownership of drivers were related to traffic accidents. Milad and Ashin [20] also used a 4-factor DBQ to compare and analyze the driving behavior of taxi and heavy truck professional drivers in Iran. The study indicated that the frequency of violations, aggressive violations and errors of taxi drivers was higher than that of truck drivers. Guého and Granie [21] developed a 6-factor structured DBQ for drivers of all ages and level of experience in France. The results also showed the link between demographic variables (age and gender), mobility (kilometers driven weekly), the DBQ scores and the involvement in an accident in the previous five years.

### Gap and research purpose

In order to reduce the huge loss of public safety and economy in traffic accidents caused by heavy semi-trailer trucks in China, it was necessary to understand the causes of driving behavior of professional drivers of heavy semi-trailer trucks in China and what kind of impact they had on traffic safety operation. DBQ was recognized as the most practical method to measure driving behavior and predict accidents. However, authors obtained diverse results from the application of DBQ due to social and cultural differences among different countries [6]. Warner et al. 2011 also reported that drivers from different countries and of different models might have different aberrant driving behaviors [22]. Therefore, it was of necessity to apply a DBQ separately for professional drivers of heavy semi-trailer trucks in China. In summary, this research aimed to expand the DBQ in a comprehensive way for Chinese professional drivers of heavy semi-trailer trucks. We also surveyed the relations among different driving behaviors, driver background information and traffic accidents. The last purpose of the research was to put forward suggestions as to validating and improving the DBQ for professional drivers of heavy semi-trailer trucks in China.

## Methods

### Materials

In order to consider as many predictors as possible, this DBQ consisted of three parts, with a large number of items. The first part was about the demographic variables and basic information of professional drivers of heavy semi-trailer trucks in China. Previous studies showed that the effects of work-rest patterns, lifestyle and payment incentives on long-haul truck drivers were closely related to driving safety [23,24]. The second part was to survey the traffic accidents involving professional drivers of heavy semi-trailer trucks in China in the past three years. Traffic accidents were defined as injuries to traffic participants and loss of vehicles or property in addition to casualties [17]. The third part was the newly developed DBQ for professional drivers of heavy semi-trailer trucks in China, which included two sub-questionnaires: the aberrant driving behavior questionnaire (DBQ) and the positive driving behavior questionnaire (PDBQ). Based on the well-developed DBQ [8,16,21,25] and existing PDBQ [9–11,26], we produced a revised DBQ for professional drivers of heavy semi-trailer trucks in China. Ultimately, a 29-item DBQ and a 7-item PDBQ were developed. Participants were asked to assess the situation described in each item of the questionnaire, and each of the item was scored based on Likert 5 (from 1 “never” to 5 “nearly all the time”).

### Participants

From August to December 2019, we had all questionnaires completed by drivers of heavy semi-trailer trucks based on their driving conditions during field survey. For the purpose of the driving behavior survey of professional drivers of heavy semi-trailer trucks in China, sample data was collected from the Hancheng Lake Service Area along Jingkun Expressway (Beijing-Kunming) in Xi’an City, Shaanxi Province, China. This service area welcomed heavy semi-trailer trucks from all parts of China, almost all of which were inter-provincial long-haul trucks. It took 5 to 10 minutes to complete a questionnaire. A professional driver of heavy semi-trailer truck was paid 5 yuan or given a towel as compensation to complete a questionnaire. A principle of anonymity and voluntariness was adopted for professional drivers to fill in the questionnaires. A total of 300 questionnaires were handed out, and 277 were collected (75.4%). Excluding incomplete questionnaires, we got 202 valid ones. This study was based on the research project of Henan Provincial Department of Transportation—Research on Operation Control Technology of Henan Expressway Based on Traffic Behavior Safety (Project No.: 2019g-2-11).

### Data analysis

Data was processed by IBM SPSS statistics 25. Given that the data would deviate from the normal distribution, we adopted a nonparametric method to verify whether each item in the questionnaire conformed to the normal distribution [6]. KMO parameters of principal component analysis, Promax rotation, and Bartlett’s test were combined to test the structure of the questionnaire. Cronbach’s Alpha was adopted to measure the reliability of the questionnaire. Correlation analysis was used to analyze driver behaviors and the influencing factors. The binary logistic regression was used to testify the relation between heavy semi-trailer truck drivers involved in traffic accidents and their driving behaviors. The predictive model was also tested.

## Results

### Descriptive statistics

The basic information of professional drivers of heavy semi-trailer trucks involved in the survey is shown in Table 1.

**Table 1:** Sample characteristics.

Attribute	Classification	Frequency (%)	Mean	SD
**1. Age**	Continuous variable (years)	-	42.13	6.91
**2. Marital status**	Single = 1	5.0	1.95	0.22
	Married = 2	95.0		
**3. Family population**	Continuous variable (people)	-	4.29	1.18
**4. Education**	Junior high school and below = 1	67.8	1.36	0.57
	High school or technical secondary school = 2	29.7		
	Junior college = 3	1.5		
	Bachelor degree and above = 4	1.0		
**5. Driving age**	Continuous variable (years)	-	14.00	6.25
**6. Monthly income**	20,000 yuan and above = 1	13.9	2.24	0.68
	1 to 20,000 yuan = 2	48.0		
	Less than 10,000 yuan = 3	38.1		
**7. Driving time per day**	Continuous variable (hours)	-	11.04	1.56
**8. Average annual mileage**	Continuous variable (ten thousand kilometers)	-	12.22	4.68
**9. Sleep time per day**	1–3 hours = 1	7.4	2.68	0.82
	4–7 hours = 2	32.2		
	7–8 hours = 3	45.0		
	9–10 hours = 4	15.3		
**10. Vehicle ownership**	Owned = 1	47.5	2.01	1.05
	Common ownership = 2	10.4		
	Company owned = 3	35.1		
	Other = 4	6.9		
**11. Bank lending**	Yes = 1	54.5	1.46	0.50
	No = 2	45.5		
**12. Preferred driving time**	1–5 a.m. = 1	12.9	2.25	0.86
	6–12 a.m. = 2	63.4		
	1–6 p.m. = 3	9.4		
	7–12 p.m. = 4	14.4		
**13. Traffic accidents in last three years**	No = 0	77.2	0.23	0.42
	At least once = 1	22.8		

### Factor analysis

IBM SPSS Statistics 25 was used to calculate the modified items and total correlation (CITC) of the DBQ (including the DBQ and the PDBQ) for professional drivers of heavy semi-trailer trucks. CITC was usually represented by average corrected inter-item total correlation (*Aiic*). Items with Aiic value less than 0.33 were excluded [20]. Every time an item was deleted, an *Aiic* calculation was conducted. In the DBQ, 5 items were deleted successively, namely 1 (*Aiic* = -0.1607), 11 (*Aiic* = 0.194), 33 (*Aiic* = 243), 2 (*Aiic* = 0.274), and 4 (*Aiic* = 272), and a total of 25 items were retained. In the PDBQ, the original 7 questions were retained. The details are shown in Table 2.

**Table 2:** Factor structure of DBQ and PDBQ.

	Title number	Dimension	Mean (SD)	Factor loading
**DBQ**	1. Risky violations (RV) *(ɑ = 0*.*73*, *Aiic = 0*.*631*, *Ev = 28*.*601%*, *Dimension’s M (SD) = 1*.*571 (0*.*547))*			
	27	Forcibly drive in or out of the line when lining up	1.36(0.728)	0.752
	23	Answer or make calls with mobile phone or on WeChat while driving	1.95(1.045)	0.700
	18	Run a red light in the middle of the night	1.50(0.748)	0.603
	13	Overtake on right lane (inner lane)	1.62(0.945)	0.540
	31	Forget to turn on signal lamp at a turning in a hurry or to get ahead of others	1.55(0.773)	0.445
	14	Whistle or use fingers toward or shout at other drivers to express dissatisfaction	1.46(0.733)	0.439
	2. Negligence/Lapses *(ɑ = 0*.*61*, *Aiic = 0*.*602*, *Ev = 8*.*267%*, *Dimension’s M (SD) = 1*. *715 (0*.*524))*			
	22	Slam on the brake under wet or other bad road conditions	1.62(0.885)	0.683
	6	Misread or misunderstand traffic signs, thus going the wrong way	2.01(0.788)	0.618
	26	Hit something that has not been noticed when backing up	1.69(0.644)	0.576
	28	Use mobile phone to read SMS, or browse web page or video while driving	1.53(0.754)	0.555
	3. Errors *(ɑ = 0*.*60*, *Aiic = 0*.*559*, *Ev = 6*.*920%*, *Dimension’s M (SD) = 1*.*529 (0*.*503))*			
	7	Wrongly estimate the time of green light, making it impossible to stop safely	1.75(0.773)	0.649
	17	Miss a highway or expressway exit as there is no time to change lanes	1.65(0.740)	0.649
	35	Have not noticed pedestrians, bicycles or electromobiles at a turning	1.34(0.621)	0.636
	36	Occupy the fast lane (overtaking lane) for a long time	1.38(0.821)	0.436
	4. Ordinary violations *(ɑ = 0*.*60*, *Aiic = 0*.*473*, *Ev = 6*.*100%*, *Dimension’s M (SD) = 1*.*798(0*.*570))*			
	3	Drive beyond the speed limit on expressways or national and provincial roads	1.77(0.698)	0.733
	16	Fail to notice traffic signs such as “give way” or “truck-no-entry”, thus entering the forbidden area	1.97(0.810)	0.614
	8	Ignore speed limit in residential areas	1.66(0.796)	0.611
**P** **DBQ**	5. Positives *(ɑ = 0*.*773*, *Ev = 46*.*812%*, *Dimension’s M (SD) = 3*.*62 (0*.*9654))*			
	10	Keep a proper following distance so as not to affect the vehicles ahead	3.99(1.366)	0.745
	25	Care about and consider whether other vehicles are affected when parking on the side of the road	3.31(1.472)	0.739
	20	Adjust speed to facilitate overtaking	3.99(1.236)	0.711
	34	Seldom use high beam so as not to disturb oncoming traffic	3.86(1.442)	0.680
	29	Try not to honk the horn to avoid affecting others	3.33(1.511)	0.611
	15	Try not to take the fast lane so as not to affect other vehicles	3.17(1.453)	0.604

Notes: Factor loadings < 0.40 not reported. *Aiic* = Average corrected inter-item total correlation. Ev = Explained variance.

Exploratory factor analysis was used to determine the dimensions of DBQ and PDBQ. The Kaizer criterion (an eigenvalue above 1.00) was used as the criterion for factor extraction. A principal component analysis (PCA) with varimax rotation and iteration was carried out. It was preliminarily confirmed that the DBQ was composed of 6 dimensions, while the PDBQ had only 1 dimension. A factor loading above 0.40 was used as a criterion for items to be retained in the DBQ dimensions [20]. As some items did not match with the target factors in the questionnaire of the DBQ, we did factor analysis again every time we removed one such item. After multiple factor analyses, 5 items were removed successively in the DBQ, namely 14, 21, 24, 9, 12, 30. Then we used the maximum variance rotation to test the communality extraction of the questionnaire items (those less than 0.33 being removed). Item 14 was removed from the PDBQ. Details are shown in Table 2. The KMO coefficients of PDBQ and DBQ were 0.822 and 0.852, respectively. The significant level of Bartlett’s sphericity test was ρ < 0.000, which showed that the questionnaire had good validity. The accumulated loads of PDBQ and DBQ were 46.684% and 49.888%, respectively. Finally, the PDBQ composed of 6 items and the DBQ composed of 17 items were determined. The internal consistency coefficients (a) of the two questionnaires were 0.773 and 0.881, respectively.

### Correlation test

The relationship among factors in the DBQ and the PDBQ is shown in Table 3. Positive driving behavior was negatively correlated with risk violations, errors and ordinary violations, respectively. It was obvious that a polite driver of heavy semi-trailer truck in China always avoided the behavior that might affect other drivers in the traffic scene, and had less traffic demand.

**Table 3:** Correlation matrix between factors of the questionnaire and total score.

	Risky violations	Negligence/Lapses	Errors	Ordinary violations
**Risky violations**	1.000	-	-	-
**Negligence/Lapses**	0.529**	1.000	-	-
**Errors**	0.508**	0.505**	1.000	-
**Ordinary violations**	0.442**	0.390**	0.329**	1.000
**Positives**	-0.179*	-0.059	-0.205**	-0.140*

* p< 0.01 (two tailed), significant correlation.

** p< 0.05 (two tailed), significant correlation.

As statistical variables might not belong to normal distribution or sequential questionnaire data, Spearman’s range correlation coefficient (Rho) was used to verify the correlation between the demographic variables and driving behavior of professional drivers of heavy semi-trailer trucks in China. At the same time, a test was conducted on whether the naming of each factor of the 17-item DBQ was reasonable, as shown in Table 4.

**Table 4:** Correlation matrix between factors of the questionnaire and total score.

Attribute	Risky violations	Negligence/Lapses	Errors	Violations	Positives
**Age**	0.026	-0.090	-0.127	0.016	-0.002
**Marital status**	-0.190**	-0.169*	-0.174*	-0.164*	0.018
**Family population**	-0.054	0.072	-0.038	-0.040	-0.025
**Education**	0.040	0.047	-0.029	0.011	0.006
**Driving age/experience**	0.057	-0.030	-0.143*	0.071	-0.014
**Average annual mileage**	0.049	0.163*	0.058	0.057	-0.110
**Monthly income**	0.027	-0.025	-0.018	0.064	0.095
**Vehicle ownership**	-0.035	-0.062	-0.045	0.050	-0.032
**Bank lending**	0.011	-0.087	-0.070	0.141*	-0.037
**Sleep time per day**	-0.293**	-0.092	-0.259**	-0.201**	0.006
**Driving time per day**	0.112	0.181*	0.025	0.108	0.075
**Preferred driving time**	-0.003	0.017	0.062	0.005	-0.075

* ρ< 0.01 (two tailed), significant correlation.

**ρ< 0.05 (two tailed), significant correlation.

### Prediction

Previous studies showed that traffic accidents were not subject to normal distribution, but Poisson distribution [6]. Therefore, binary logistic regression analysis was adopted to predict the occurrence of traffic accidents. We used forward step to filter the variable, and finally got a full model, as shown in Table 5.

**Table 5:** Prediction of crashes over previous three years.

Number	Variable	B	Std. Err	WALD	Significance	OR
**1**	Negligence/Lapses	0.830	0.334	6.174	0.013	2.293
**2**	Preferred driving time	-	-	8.366	0.039	-
	Preferred driving time (1)	2.133	1.054	4.097	0.043	8.437
	Preferred driving time (2)	1.460	0.730	4.004	0.045	4.307
	Preferred driving time (3)	1.374	0.551	6.204	0.013	3.949
**3**	Constant	-2.950	0.704	17.546	0.000	0.052

Ref.: ‘Reference category’; ‘**’ < 0.05; ‘***’ < 0.01. IRR = Incidence rate ratios.

Overall model χ^2^ (4, N = 202) = 19.78, p < 0.001; Hosmer and Lemeshow test χ^2^ (7) = 2.634, p = 0.917; Nagelkerke r^2^ = 0.142 and Cox and Snell γ^2^ = 0.093.

## Discussion

In China, traffic accidents caused by heavy semi-trailer trucks were serious. The most common aberrant driving behaviors of heavy semi-trailer truck drivers in China were “misreading or misunderstanding traffic signs, thus going the wrong way”, followed by “failing to notice traffic signs such as ‘give way’ or ‘truck-no-entry’, thus entering the forbidden area” and “answering or making calls with mobile phone or on WeChat while driving”. The error with the lowest frequency was “having not noticed pedestrians, bicycles or electromobiles at a turning”. This was inconsistent with previous researches on non-professional drivers and professional drivers. Most researches found that the frequency of “speeding” or “using mobile phones while driving” was the highest among drivers’ aberrant driving behaviors [6,17,26]. This might be due to the vast territory of China. Heavy semi-trailer truck drivers drove across multiple provinces for transportation, making them work for long hours and drive unfixed routes. They were often alone in new places. When a driver was faced with complex traffic and road conditions, it could easily lead to more errors. In addition, due to the heavy weight, large volume, limited speed, long body and large blind area of vision of a heavy semi-trailer truck, drivers were more careful when taking turnings. The improvement of road traffic conditions was beneficial to the establishment of drivers’ sense of safety. Therefore, it was also effective to reduce traffic accidents by improving the conditions of road traffic.

Positive driving behavior in the lowest frequency among heavy semi-trailer truck drivers in China was “trying not to take the fast lane so as not to affect other vehicles”; the most common positive driving behaviors were “keeping a proper following distance so as not to affect the vehicles ahead” and “adjusting speed to facilitate overtaking”. The frequency of positive driving behaviors of drivers of heavy semi-trailer trucks in China was lower than that of general heavy truck drivers in previous studies [6]. This might be due to the general time limit of cargo transportation, and drivers of heavy semi-trailer trucks generally occupied the fast lane to save time (reward) and improve efficiency. In addition, most of the heavy semi-trailer trucks in China were privately owned. With low education level and weak safety awareness, most drivers of such trucks were attached to logistics transportation enterprises and received less safe driving education and training. This led to the ordinary violations or errors of the heavy semi-trailer trucks in China, such as frequent use of mobile phones and insufficient following distance for business or social needs.

The results revealed that the frequency of risk violations, errors and ordinary violations of Chinese heavy semi-trailer truck drivers was lower when the frequency of positive driving behaviors was higher. This was consistent with the previous studies [6]. It might be due to the fact that positive driving behavior positively affected the driving style of drivers [9]. Positive driving behavior could also produce a traffic atmosphere which helped the driver to reduce aberrant driving behavior to a minimum. Especially for the drivers of heavy semi-trailer trucks, most of them were engaged in long-distance freight business that demanded high work intensity and frequent driving on highways. Safe driving style might reduce the influence of aberrant driving behaviors. However, in China, driving training schools, logistics and freight enterprises mainly offered drivers with safety education courses on how to improve driving skills, and there was no course for positive driving behavior. Therefore, the traffic management department should pay attention to the traffic propaganda of drivers’ positive driving behavior. Traffic safety was not only to alert drivers to traffic accidents and conflicts, but also advocate a positive traffic culture rather than fear of danger. However, positive driving behavior of heavy semi-trailer truck drivers in China was not related to negligence/lapses, which was inconsistent with the previous research conclusions [6]. This was possibly because negligence/lapses were caused by unfamiliarity with the road, traffic rules, and traffic signs [27]. Heavy truck drivers with more positive driving behaviors mainly had less aggressive behaviors, and all of them abided by traffic laws and regulations.

This paper analyzed the correlation between the background variables of professional drivers of heavy semi-trailer trucks and their driving behaviors. It was found that risk violations and ordinary violations of married heavy semi-trailer truck drivers were in low frequency, which was consistent with previous studies. Results also showed that married drivers had low frequency of negligence/lapses and errors, which was inconsistent with previous studies [6]. It might result from the fact that single drivers were highly possible to take risks or drive for kicks. Therefore, their risk violations and other violations were more frequent. In addition, through the correlation analysis between drivers’ background variables, it could be found that the driving age of single heavy semi-trailer truck drivers was relatively low, possibly due to the lack of driving experience, which led to the high frequency of errors. It was also revealed that the higher the driving age was, the less errors heavy semi-trailer truck drivers made. The study found no evidence that the age of heavy semi-trailer truck drivers in China was related to each driving behavior, which was inconsistent with previous studies [18].

The fatigue problem of drivers operating vehicles has always been a research hotspot. It was found that the sleep time of heavy semi-trailer truck drivers in China was negatively correlated with risk violations, errors and ordinary violations, respectively. It might be due to the negative effects of sleep time on drivers’ psychological and physiological states [18]. In general, driving time and sleeping time directly affected driver fatigue, and the daily level of fatigue was directly proportional to aberrant driving behaviors [28]. Drivers with good sleep conditions had fewer ordinary violations and errors, and driver fatigue was directly related to the frequency of errors [10]. Such drivers were less likely to have traffic accidents. It also showed that the average annual driving mileage and daily driving time of heavy semi-trailer truck drivers were positively correlated with negligence/lapses. Useche et al. 2017 found that as the daily driving time increased, the probability of traffic accidents raised [29]. Therefore, the traffic safety management department should formulate supervision measures, and reasonably lay down restrictive measures based on the continuous driving time and rest frequency of heavy semi-trailer truck drivers.

With the prediction of accident factors through the binary logistic regression model, it was found that the variables related to traffic accidents were negligence/lapses and appropriate driving time. The prediction model showed that the probability of traffic accidents increased by 2.293 times for each unit increase of negligence/lapses of heavy semi-trailer truck drivers, which was consistent with previous studies [6]. The probability of traffic accidents caused by heavy semi-trailer truck drivers apt to drive during 1–5 a.m. was 8.437 times higher than that of those apt to drive during 7–12 p.m. Drivers apt to drive during 7–12 a.m. were 4.307 times more likely to have traffic accidents than those apt to drive during 7–12 p.m. Drivers apt to drive during 1–6 p.m. were 3.949 times more likely to have traffic accidents than those apt to drive during 7–12 p.m. It could be attributed to the fact that 1–5 a.m. was a period during which drivers were most exhausted. Therefore, driving vehicles in that period was prone to accidents, and causing major casualties. Hence, the *Safety Management Code for Road Passenger Transport Enterprises* and other traffic laws prohibited large passenger transport vehicles from operating on the highway during 2–5 a.m. However, during this period, heavy semi-trailer trucks were not prohibited from running on the highway. It was suggested that the transportation department strengthen the supervision of heavy semi-trailer trucks and other heavy vehicles operating during 1–5 a.m., and strictly inspect traffic ordinary violations such as fatigue driving.

## Supporting information

S1 Table Driver Behavior Questionnaire (DBQ). (DOCX)

S1 Data (SAV)
