# Supplementary material for: Driver behaviour and traffic accident involvement among professional heavy semi-trailer truck drivers in China
Source: PLoS One. 2021 Dec 2;16(12):e0260217. doi: 10.1371/journal.pone.0260217 (PMC8638885; doi:10.1371/journal.pone.0260217)
Supplement: S1 Table — (DOCX) [file pone.0260217.s001.docx]

**Supporting information**

**S1 Table. Driver Behavior Questionnaire (DBQ)**.

1. Age ; 2. Marital status ; 3. Family population ;

4. Education ;

A. Junior high school and below; B. High school or technical secondary school;

C. Junior college; D. Bachelor degree and above

5. Driving age ;

6. Monthly income ; A. 20,000 yuan and above; B. 1 to 20,000 yuan; C. Less than 10,000 yuan

7. Driving time per day ; 8. Average annual mileage ;

9. Sleep time per day ; A. 1-3 hours; B. 4-7 hours; C. 7-8 hours; D. 9-10 hours = 4

10. Vehicle ownership ; A. Owned; B. Common ownership ; C. Company owned ; D. Other

11. Bank lending ; A. Yes; B. No

12. Preferred driving time ; A. 1-5 a.m. B. 6-12 a.m. C. 1-6 p.m. = 3 D. 7-12 p.m.

13. Traffic accidents in the last three years ; A. No = 0; B. At least once

| **NO.** | **Item** | **Never/Occasionally/Sometimes/Often/Nearly all the time** | | | | |
| --- | --- | --- | --- | --- | --- | --- |
| 3 | Drive beyond the speed limit on expressways or national and provincial roads | 1 | 2 | 3 | 4 | 5 |
| 6 | Misread or misunderstand traffic signs, thus going the wrong way | 1 | 2 | 3 | 4 | 5 |
| 7 | Wrongly estimate the time of green light, making it impossible to stop safely | 1 | 2 | 3 | 4 | 5 |
| 8 | Ignore speed limit in residential areas | 1 | 2 | 3 | 4 | 5 |
| 10 | Keep a proper following distance so as not to affect the vehicles ahead | 1 | 2 | 3 | 4 | 5 |
| 13 | Overtake on right lane (inner lane) | 1 | 2 | 3 | 4 | 5 |
| 14 | Whistle or use fingers toward or shout at other drivers to express dissatisfaction | 1 | 2 | 3 | 4 | 5 |
| 15 | Try not to take the fast lane so as not to affect other vehicles | 1 | 2 | 3 | 4 | 5 |
| 16 | Fail to notice traffic signs such as “give way” or “truck-no-entry”, thus entering the forbidden area | 1 | 2 | 3 | 4 | 5 |
| 17 | Miss a highway or expressway exit as there is no time to change lanes | 1 | 2 | 3 | 4 | 5 |
| 18 | Run a red light in the middle of the night | 1 | 2 | 3 | 4 | 5 |
| 20 | Adjust speed to facilitate overtaking | 1 | 2 | 3 | 4 | 5 |
| 22 | Slam on the brake under wet or other bad road conditions | 1 | 2 | 3 | 4 | 5 |
| 23 | Answer or make calls with mobile phone or on WeChat while driving | 1 | 2 | 3 | 4 | 5 |
| 25 | Care about and consider whether other vehicles are affected when parking on the side of the road | 1 | 2 | 3 | 4 | 5 |
| 26 | Hit something that has not been noticed when backing up | 1 | 2 | 3 | 4 | 5 |
| 27 | Forcibly drive in or out of the line when lining up | 1 | 2 | 3 | 4 | 5 |
| 28 | Use mobile phone to read SMS, or browse web page or video while driving | 1 | 2 | 3 | 4 | 5 |
| 29 | Try not to honk the horn to avoid affecting others | 1 | 2 | 3 | 4 | 5 |
| 31 | Forget to turn on signal lamp at a turning in a hurry or to get ahead of others | 1 | 2 | 3 | 4 | 5 |
| 34 | Seldom use high beam so as not to disturb oncoming traffic | 1 | 2 | 3 | 4 | 5 |
| 35 | Have not noticed pedestrians, bicycles or electromobiles at a turning | 1 | 2 | 3 | 4 | 5 |
| 36 | Occupy the fast lane (overtaking lane) for a long time | 1 | 2 | 3 | 4 | 5 |
